# Long-Term Mortality Outcomes According to the Frequency of Right Ventricular Pacing in Veterans

**DOI:** 10.4061/2010/310768

**Published:** 2010-05-05

**Authors:** Brent C. Lampert, Hans J. Moore, Richard L. Amdur, Pamela E. Karasik, Brian M. Lewis, Steven N. Singh, Ross D. Fletcher

**Affiliations:** ^1^School of Medicine, Georgetown University, Washington, DC 20057, USA; ^2^United States Department of Veterans Affairs, Washington Veterans Affairs Medical Center, Washington, DC 20422, USA; ^3^School of Medicine, George Washington University, Washington, DC 20037, USA; ^4^School of Medicine, Howard University, Washington, DC 20059, USA

## Abstract

*Background*. Right ventricular pacing (RVP) has been associated with adverse outcomes, including heart failure and death. Minimizing RVP has been proposed as a therapeutic goal for a variety of pacing devices and indications. *Objective*. Quantify survival according to frequency of RVP in veterans with pacemakers. *Methods*. We analyzed electrograms from transtelephonic monitoring of veterans implanted with pacemakers between 1995 and 2005 followed by the Eastern Pacemaker Surveillance Center. We compared all cause mortality and time to death between patients with less than 20% and more than 80% RVP. *Results*. Analysis was limited to the 7198 patients with at least six trans-telephonic monitoring records (mean = 21). Average follow-up was 5.3 years. Average age at pacemaker implant was significantly lower among veterans with <20% RVP (67 years versus 72 years; *P* < .0001). An equal proportion of deaths during follow-up were noted for each group: 126/565 patients (22%) with <20% RVP and 1113/4968 patients (22%) with >80% RVP. However, average post-implant survival was 4.3 years with <20% RVP versus 4.7 years with >80% RVP (*P* < .0001). *Conclusions*. Greater frequency (>80%) of RVP was not associated with higher mortality in this population of veterans. Those veterans utilizing <20% RVP had a shortened adjusted survival rate (*P* = .0016).

## 1. Introduction

Right ventricular apical pacing (RVP) is commonly employed, but concerns have been raised suggesting that it is associated with worsened mortality in the setting of cardiomyopathy. Several trials have found an association between more frequent RVP and adverse effects, such as atrial fibrillation and congestive heart failure [1–4]. Dual-chamber pacing can improve quality of life in the absence of atrioventricular block, which requires less frequent RVP; however it does not reduce the rate of stroke or death [5–7]. As interest has grown in using biventricular pacing to reduce mortality and symptoms in certain patients with left ventricular systolic dysfunction, the advisability of frequent RVP has been questioned [8]. Minimizing right ventricular pacing has even been proposed for unselected pacemaker patients, regardless of left ventricular function [9]. Although multiple studies have addressed mortality, either they have included limited numbers of subjects or had relatively short followup intervals [1, 5, 6]. The present study from a large national cohort of veterans retrospectively evaluates whether the frequency of right ventricular pacing was associated with shortened survival or altered all-cause mortality during long-term followup.

## 2. Methods

The Eastern Pacemaker Surveillance Center is one of two national Veterans Administration centers established for remote telephonic monitoring. It has served Veterans for 25 years, maintaining a large registry of transtelephonic monitoring records (TTMs) and outcomes. Quality assurance analysis of deidentified data from this population was used to assess for effects of frequent RVP, and potential need for reprogramming pacemakers to minimize RVP. From this registry of over 66,000 patients, we identified those with permanent pacemakers which had active right ventricular leads implanted between January 1, 1995, and December 31, 2005. This group was then limited to those with a minimum number of TTM followups, who had either a very high (>80%) or very low (<20%) frequency of RVP. Frequency was determined by the TTM recordings (typically Lead I) which lasted 30 seconds before and 15 seconds during magnet application. The percent of paced ventricular complexes on each TTM was noted. The values were averaged for each patient and used as a representation of that patient's frequency of RVP.

**Figure 1:**
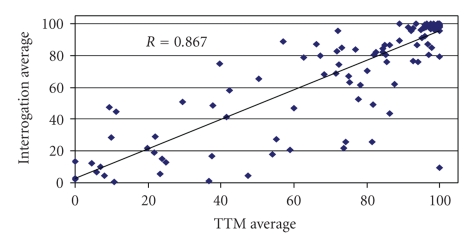
Number of TTMs versus Percentage RVP from interrogation. Correlation coefficient is for entire group. Values analyzed were either <20% or >80%, which fell closer to the line of identity. (RVP = right ventricular pacing. TTMs = transtelephonic monitor reports.)

We reviewed records of 174 patients from the Washington Veterans Affairs Medical Center with 3 or more TTMs to determine the minimum number of prior TTMs needed. The average frequency of TTM-derived RVP was compared to at least 2 independent records of frequency of RVP obtained from implanted pacemaker generated data logs from office-based pacemaker interrogation. A minimum of 6 TTM-derived RVP values (Table 1 and Figure 1) correlated sufficiently with the data log estimates (*R* = 0.867) so that the current analysis required veterans with at least 6 TTMs. 

 Our group had previously analyzed outcomes for very high and very low frequency RVP based on those with less than 20% right ventricular pacing (<20% RVP) and those with greater than 80% (>80% RVP) [10] excluding patients with atrial single chamber, biventricular pacemakers, and implanted pacemaker defibrillators. These allocations were considered reasonable and they were used in this study. Testing for other allocations was not performed. Survival was assessed from the Eastern Pacemaker Surveillance Center records, and verified through Veterans Affairs related data sources for all subjects in October 2006.

## 3. Statistical Analysis

The primary endpoints were all-cause mortality and post pacemaker implant survival, measured as the time from pacemaker insertion to death. We examined univariate relationships between predictors (patient and pacemaker characteristics) and outcomes using Kaplan-Meier analysis (Proc Lifetest in SAS version 9.1). Multivariate relationships were examined using Cox regression (Proc Phreg in SAS version 9.1). 

## 4. Results

**Figure 2:**
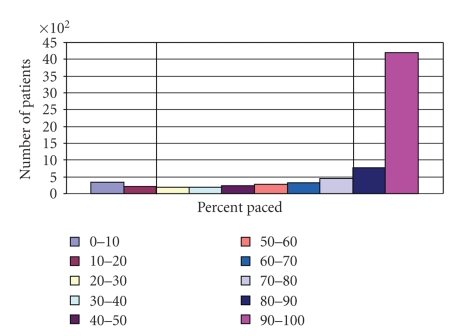
Bar graph showing patient distribution by percent pacing. Allocation of patients to percent-paced decile based upon frequency of RV pacing detected by transtelephonic monitoring records. Patients in the first and second deciles, shown below vertical line at 20%, were compared to patients in the ninth and tenth deciles, shown above vertical line at 80%.

We identified 7198 patients from the Eastern Pacemaker Surveillance Center registry with six or more TTMs (Mean = 21 TTMs) during the 11-year time period with either <20% RVP (*n* = 565) or >80% RVP (*n* = 4968). This represented 77% of all patients with at least 6 TTMs (Figure 2). Significant differences in the types of pacemakers and indications for pacing were noted between the two groups (Table 2). The average duration of post-implant followup was 5.1 ± 2.5 years in those with <20% RVP and 5.3 ± 2.4 years from time of pacemaker implantation in those with >80% RVP (*P* = .062). 

 Correlation of clinical variables with mortality outcomes was analyzed in a regional group of 174 patients at the Washington Veterans Affairs Medical Center (Table 3). This group showed minor differences from the larger cohort in terms of age, distribution between <20% RVP and >80% RVP, and rate responsiveness. Clinical variables evaluated included the presence of coronary artery disease, diabetes, dyslipidemia, heart failure, hypertension, and medications (angiotensin-converting enzyme inhibitors, angiotensin receptor blockers, aspirin, beta-blockers, calcium channel blockers, diuretics, and statins). There were no significant differences in these clinical variables in this subgroup, between patients with <20% RVP and >80% RVP. However we cannot with a high confidence level conclude that other clinical variables did not impact outcomes in the larger cohort. 

Average age at time of pacemaker implant was significantly lower in the group who had <20% RVP versus >80% RVP (67 versus 72 years; *P* < .0001). The overwhelming majority of patients were men (98% in both groups). Single-chamber pacemakers were present in 32% of patients who had <20% RVP and in 18% of patients who had >80% RVP (Chi-square = 80.00, *P* < .001). Sinus node dysfunction was the pacing indication slightly more often in those patients with <20% RVP (66%), compared to those patients with >80% RVP (56%); whereas atrioventricular node block was the pacing indication slightly less often in those patients with <20% RVP (34%) compared to those patients with >80% RVP (44%). 

**Figure 3:**
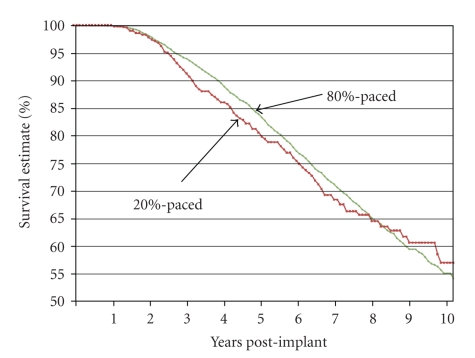
Survival function for <20% versus >80% RVP. Although Kaplan-Meier analysis did not find a significant difference between these groups when they were examined without covariates, Cox regression analysis controlling for age at implant, pacemaker type, and rate responsiveness showed that overall survival was better for those veterans with >80% RVP (*P* = .0016). Lower age, dual-chamber pacemaker, and rate responsiveness were associated with better survival. Most of the difference in risk occurs between years 3 and 8 post implant. There is little difference prior to year 2.

**Figure 4:**
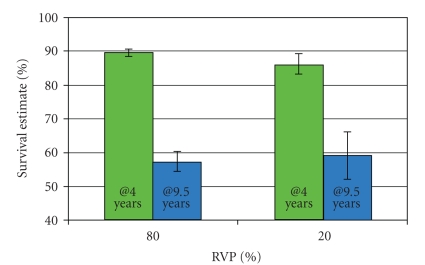
Survival outcomes at 4 years and 9.5 years post implant. At 4 years post implant, 90% of patients with >80% RVP (95% CI, 89%–91%) remain alive, compared with 86% of patients with <20% RVP (95% CI, 83%–89%) (*P* < .05). However, at 9.5 years post implant, the survival rates were 57% (95% CI, 55%–60%) for patients with >80% RVP and 59% (95% CI, 52%–66%) for patients with <20% RVP, which were not significantly different. (RVP = right ventricular pacing.)

Kaplan-Meier analysis (Table 4 and Figure 3) found that the patients with <20% RVP compared to patients with >80% RVP did not differ in percent of patients who survived (22.3% versus 22.4%; Chi-square = 0.75; *P* = .39) or in survival time (75% survival occurred at 6.1 years (95% CI = 5.3–6.7 years) for the 20% RVP group; and at 6.4 years, (95% CI = 6.2–6.7 years) in the >80% RVP group). Survival was also assessed at two separate time points post implant: 4 years and 9.5 years post implant. Significantly more patients with >80% RVP were alive at 4 years; however, at 9.5 years survival rates were not significantly different (Figure 4).

 Pacemaker type (single versus dual), age at implant, and rate responsiveness all had significant univariate relationships with survival time (all *P* < .001). There were a small percentage of patients in both groups without rate responsiveness. Rate responsiveness was an independent predictor of better survival (*P* = .01). Since the <20% RVP versus >80% RVP patients differed in age at implant, pacemaker type, and percent with rate responsiveness, it was necessary to do a multivariate analysis using Cox regression in order to determine the independent effect of <20% RVP versus >80% RVP after accounting for age, pacemaker type, and rate responsiveness. This analysis found that <20% RVP versus >80% RVP, age at implant, and pacemaker type together were significantly related to survival time (Chi-square = 333.40; *P* < .0001), and that each of these variables independently had a significant impact on survival time (Table 5). In multivariate analysis, patients with >80% RVP lived significantly longer than patients with <20% RVP (Chi-square = 9.92; *P* = .0016).

 Having <20% RVP in comparison to >80% RVP was associated with a 36% higher likelihood of death during the followup period (*P* = .0016), after accounting for the effects of all other covariates. Having a single-chamber pacemaker (versus dual chamber) was associated with a 28.6% higher likelihood of death during the followup period (*P* < .001). Absence of rate responsiveness raised the risk of death by 29.6% (*P* = .01). Each year of age was associated with a 6.0% higher likelihood of death during the 5.3-year average followup period (*P* < .0001). 

In order to more closely examine the concurrent effects of both percent pacing (<20% RVP versus >80% RVP) and pacemaker type (single chamber versus dual chamber), we coded the four possible combinations of these 2 variables: (1) <20%: single, (2) <20%: dual, (3) >80%: single, and (4) >80%: dual; then we completed both Kaplan-Meier and Cox regression analyses. In the Kaplan-Meier analysis, the group variable of percent pacing and pacemaker type (with 4 levels) had a significant impact on survival (Chi-square = 41.36; *P* < .0001). Patients with <20% RVP and single-chamber pacemakers had both the highest percentage of patients who died (30.8%) and the shortest time to death (25% died by 4.5 years post implant). Patients with >80% RVP and single-chamber pacemakers also had a high percentage who died (30.5%), and a moderate survival time (25% died by 5.5 years). The two groups with dual-chamber pacemakers lived longer (25% died by 6.7 years for both <20% RVP and >80% RVP) and fewer died (18.1% and 20.8% for <20% RVP and >80% RVP, resp.; Table 6). In the Cox regression analysis with age and rate responsiveness as covariates, the percent pacing and pacemaker type variable (4 levels) together with age at implant and rate responsivity were strongly related to survival (Chi-square = 332.29; *P* < .0001). The age at implant, rate responsiveness, and the combined variable (percent pacing and pacemaker type) made significant contributions to the regression equation (Table 7). 

**Figure 5:**
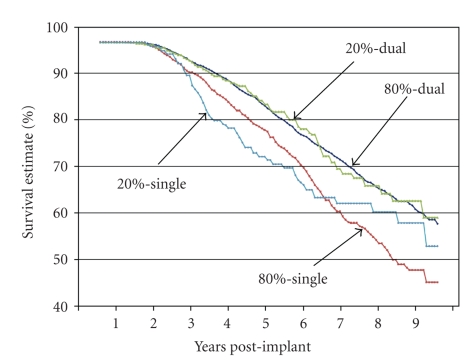
Survival function for 4 groups. The group variable, percent pacing, and pacemaker type had a significant impact on survival. Throughout the followup period, patients with dual-chamber pacemakers were least likely to die, regardless of whether they had <20% or >80% pacing. Patients with <20% and single-chamber pacemakers had both the highest percentage of patients who died (30.8%) and the shortest time to death (25% died by 4.5 years post implant). Patients with >80% and single-chamber pacemakers also had a high percentage, who died (30.5%), and a moderate survival time (25% died by 5.2 years).

 Examination of the survival curves (Figure 5) shows that during the post-implant followup period, patients with dual-chamber pacemakers were least likely to die, regardless of whether they had <20% RVP or >80% RVP.

## 5. Discussion

Our study examined the relationship between the amount of time the right ventricle is paced and subsequent mortality. We evaluated patients with an indication for anti-bradycardia pacing due to either sinus node dysfunction or AV block. Because there are inherent difficulties in controlling percent of right ventricular pacing, this retrospective analysis allowed selection of patients with a calculated frequency of RVP. We wanted the groups to have different treatment effects (either mostly paced or mostly not paced). The <20% RVP and >80% RVP cutoffs, which our group has previously used, were predetermined to allocate large groups with either a relatively low level of pacing or a relatively high level of pacing. Using these two extremes might allow better detection of a pacing effect. We did not use cutoffs of 0% RVP and 100% RVP, since far too few patients would be included in the analysis. Nor were cutoffs such as <50% RVP and >50% RVP used, because these might not discriminate pacing effect: patients in the low-pacing group could have up to 49% pacing, whereas those in the high-pacing group could have as little as 51% pacing. 

 We found that, despite being significantly older at the time of pacemaker insertion, the group that paced more frequently did not have a higher incidence of death. When survival was assessed for the overall group, and at two additional time points, more frequent right ventricular pacing did not shorten survival. The higher frequency of dual-chamber pacing in the >80% RVP group may have contributed to this difference. Despite the apparent survival advantage with more pacing at 4 years, longer followup suggests that this advantage is lost by 9.5 years. The results of even longer followup are not known. Furthermore, of those who died, the <20% RVP group had a significantly shorter survival following pacemaker implant. The suboptimal physiologic effects of right ventricular pacing therefore do not appear to lead to a worse mortality outcome. It appears that, in patients with an appropriate indication for pacemaker therapy, an increased frequency of right ventricle pacing will not hasten death. This should be reassuring to both patients with pacemakers and their physicians. 

 The physiologic effects of right ventricular pacing are well known. These can include atrioventricular dissociation, as well as valvular regurgitation leading to atrial enlargement and remodeling, and ventriculoatrial conduction, which may predispose patients to atrial fibrillation [4]. Additionally, ventricular dyssynchrony may alter cardiac hemodynamics contributing to ventricular remodeling and heart failure [4, 11]. Whether these physiologic changes ultimately effect mortality is uncertain. For instance, in the DAVID trial, dual-chamber pacing leads to worse outcomes, which was attributed to unnecessary right ventricular pacing [3]. However, several large randomized trials comparing right ventricular pacing with modalities that more closely mimic physiologic pacing have demonstrated only minimal differences in outcomes and no difference in mortality, but none of these studies followed as large of a cohort for as long as in our study [5–7]. For example, the Mode Selection Trial (MOST) in Sinus Node Dysfunction had 2568 patients with a mean follow-up of 3 years, and showed that dual-chamber pacing reduced the risk for atrial fibrillation and signs and symptoms of heart failure, but did not improve stroke-free survival [5]. In addition, Connolly et al. compared patients receiving traditional right ventricular pacing with “physiologic” pacing (atrial and atrioventricular) and found that physiologic pacing provided no significant benefit over ventricular pacing for the prevention of stroke or death due to cardiovascular cause [6]. The Pacemaker Selection in the Elderly study investigators found that dual-chamber pacing provided no significant difference in quality of life over ventricular pacing in those with atrioventricular block [7]. Furthermore, one study has demonstrated that patients with atrial fibrillation who undergo AV nodal ablation and pacemaker implantation with subsequent 100% ventricular pacing have no difference in long-term survival compared with patients who are not paced and only medically rate controlled [12]. A study of outcomes of atrial pacing compared to dual-chamber pacing in a large cohort of Swedish patients paced for sick sinus syndrome showed no difference in the standardized mortality ratios for all-cause mortality [13]. In our study, atrial pacemakers were excluded, 43% were paced for atrioventricular block, and 83% had dual-chamber pacemakers. Finally, certain patients with hypertrophic and dilated cardiomyopathies have in fact seen clinical benefit from right ventricular pacing in small studies [14, 15]. 

Despite absence of conclusive evidence demonstrating a worse mortality outcome, minimizing right ventricular pacing has become a therapeutic goal in pacemaker patients [9]. New pacing algorithms have been developed to minimize right ventricular pacing in dual-chamber pacemakers [16]. In our study, where TTMs were conducted while patients were stationary, rate responsiveness, which has the potential to cause patients in the <20% RVP group to be paced even more often, was associated with better survival. There have been proposals to use alternative pacing sites such as left ventricular or biventricular pacing in patients without heart failure [17, 18]. These approaches are not only more costly, but also technically challenging and may contribute to a higher procedural risk or need for repeat procedures. 

 In addition to the primary endpoints, this study validated that when substantial transtelephonic monitoring data is available it has a high correlation as a surrogate for actual percentage of ventricular pacing. To our knowledge, this had not been previously demonstrated. This could have implications on future study designs and open new opportunities for research on pacing therapy. Furthermore, it was observed that, among Eastern Pacemaker Surveillance Center patients with six or more TTMs, only 23% of patients received right ventricular pacing between 20% and 80% of the time. This demonstrates that patients in our study tend to fall at the extremes of being frequently paced (>80% RVP) or minimally paced (<20% RVP) while at rest for TTM recording, which could reflect not only pacing indication but also pacemaker programming. A comparison of the extremes was made; the effects of right ventricular pacing in the intermediate group with >20% RVP but <80% RVP were not assessed.

## 6. Limitations

Our observations and conclusions should be evaluated recognizing the inherent limitations of a retrospective study design. Differences in pacemaker indication and pacemaker type between the two groups may have contributed to better survival despite older age. Although the regional group analyzed did not show any statistically significant difference in comorbidities or medications used, it did differ slightly from the overall cohort. However, these are not large differences: the regional group is slightly older, has slightly more single-chamber pacing in the 20% RVP group, and slightly less in the 80% RVP group, and has slightly more rate-responsive pacing in the >80% RVP group and slightly less in the <20% RVP group. Regarding the primary survival analysis, it is possible that unrecognized differences in care or patient characteristics account for the lack of a difference in observed survival; however this finding is similar to that of a Swedish study comparing AAI pacing to DDD pacing [13]. The actual site of pacing within the right ventricular could not be verified. Furthermore, while we were able to demonstrate the use of TTM data as a reasonable surrogate for more comprehensive measures of overall percent RV pacing, it is not a perfect representative. It is notable that the group with right ventricular pacing greater than 80% of the time had the ostensible disadvantage of being significantly older at the time of pacemaker insertion. The vast majority of patients were male, and these results may not be applicable to female pacemaker patients. 

## 7. Conclusions

In our study more frequent right ventricular pacing was not followed by an increased or earlier mortality in this unselected veteran population. Following pacemaker implantation, multiple variables impacted mortality outcomes. Kaplan-Meier analysis comparing survival difference between all patients with <20% right ventricular pacing (22.3%) compared to those with >80% right ventricular pacing (22.4%) showed no difference (*P* = .39). When controlled for age at implant, type of pacemaker, and rate responsiveness, more frequent right ventricular pacing was associated with overall 36.2% higher likelihood of survival during five years of followup (*P* = .0016). Most of the difference in survival occurs during years 3 through 8 post implant. There was little difference prior to year 2. 

 Despite the potential negative physiologic effects of right ventricular pacing that have been previously demonstrated in select patient groups, our findings suggest that right ventricular pacing per se does not have a deleterious effect on survival. Review of this large clinical database suggests that less frequent right ventricular pacing does not decrease mortality, and that more frequent right ventricular pacing does not increase mortality in an unselected veteran population. Thus from a quality assurance perspective, there does not appear to be a need for reprogramming all patients to minimize the frequency of right ventricular pacing. Large prospective or case-controlled studies would be needed to validate these findings. 

## Figures and Tables

**Table 1:** TTM validation for % right ventricular pacing.

Number of TTM values	Number of patients	Correlation
≥3	174	*R* = 0.825
≥6	143	*R* = 0.867
≥9	124	*R* = 0.870
≥12	112	*R* = 0.866

TTMs: transtelephonic monitor reports.

**Table 2:** Study findings.

	<20% RVP	>80% RVP	
Number of patients	565	4968	*P* = .0016
Age at implant (*years*)	66.67	72.10	*P* < .0001
Male	552 (98%)	4889 (98%)	*P* = ns
Rate responsiveness	516 (91%)	4732 (95%)	*P* = .01
Pacemaker type:			
Single chamber	182 (32%)	887 (18%)	*P* = .0003
Dual chamber	383 (68%)	4081 (82%)	
Indication:			
Sinus node dysfunction	373 (66%)	2782 (56%)	*P* < .01
Atrioventricular block	192 (34%)	2186 (44%)	
Average followup (*years) *± *SE *	5.1 ± 2.5	5.3 ± 2.4	*P* = .062
Deaths	126 (22.3%)	1113 (22.4%)	*P* = .39
Average time to death (*years*) (95% confidence interval)	4.34 (3.98–4.70)	4.72 (4.60–4.84)	*P* < .0001

RVP = right ventricular pacing.

**Table 3:** Clinical characteristics in Washington, DC VAMC subgroup.

	<20% RVP	>80% RVP	
Number of patients	26	148	
Age at implant	73.1	74.5	
Rate responsiveness	23 (88%)	146 (99%)	*P* = .0244
Pacemaker type:			
Single chamber	10 (38%)	22 (15%)	*P* = .0105
Dual chamber	16 (62%)	126 (85%)	
Coronary artery disease	9 (34.6%)	71 (48.0%)	*P* = .2862
Diabetes mellitus	8 (30.8%)	48 (32.4%)	*P* = 1.0000
Dyslipidemia	15 (57.7%)	76 (51.4)	*P* = .6713
Heart failure	4 (15.4%)	33 (22.3%)	*P* = .6042
Hypertension	19 (73.1%)	113 (76.4%)	*P* = .8041
ACE/ARB	14 (53.8%)	87 (58.8%)	*P* = .6707
Aspirin	13 (50%)	73 (49.3%)	*P* = 1.0000
Beta-blocker	12 (46.2%)	62 (41.9%)	*P* = .8301
Calcium channel blocker	4 (15.4%)	26 (17.6%)	*P* = 1.0000
Diuretic	13 (50%)	72 (48.6%)	*P* = 1.0000
Statin	15 (57.7%)	65 (43.9%)	*P* = .2078

ACE/ARB = Angiotensin-converting enzyme inhibitor and/or Angiotensin receptor blocker.

RVP = right ventricular pacing.

**Table 4:** Kaplan-Meier results for <20% versus >80% right ventricular pacing.

Pacing	Total *n*	*n* Died	Censored	% Died	25th %-ile	95% CI	95% CI
					for survival (years)	lower bound	upper bound
<20%	565	126	439	22.3	6.12	5.25	6.72
>80%	4972	1113	3859	22.4	6.39	6.17	6.66

Total	5537	1239	4298	22.4			

**Table 5:** Cox regression analysis using <20% versus >80% right ventricular pacing, single versus dual pacemaker type, simple versus rate responsive, and age as separate variables.

Variable	DF	Parameter estimate	SE	Chi-square	*P*	Hazard ratio
<20% (versus >80%)	1	0.309	0.098	9.92	.0016	1.362
Single (versus dual)	1	0.252	0.069	13.26	.0003	1.286
Simple (versus rate responsive)	1	0.259	0.101	6.58	.01	1.296

Age	1	0.059	0.004	248.63	<.0001	1.060

**Table 6:** Kaplan-Meier results for 4 groups.

Pacing	Total *n*	*n* Died	Censored	% Died	25th %-ile	95% CI	95% CI
					for survival (years)	lower bound	upper bound
<20% + single	182	56	126	30.77	4.46	3.81	6.00
<20% + dual	376	68	308	18.09	6.66	6.03	7.69
>80% + single	839	256	583	30.51	5.51	5.15	6.06
>80% + dual	4080	847	3233	20.76	6.70	6.38	7.02

Total	5477	1227	4250	22.40			

RVP = right ventricular pacing.

**Table 7:** Cox regression results using age, rate responsiveness, and 4-group pacing variable.

Variable	DF	Parameter Estimate	SE	Chi-square	*P*	Hazard ratio
Pacing + type	1	.189	0.036	27.90	<.0001	1.208
Simple versus rate responsive	1	.275	.100	7.52	.006	1.316

Age	1	.059	0.004	260.79	<.0001	1.061

Pacing + type is the composite variable of <20% or >80% pacing and single- or dual-chamber pacemaker type.

## Abbreviations

RVP: Right ventricular pacing
TTM: Transtelephonic monitoring record.
